# Evolution of thrombolytic therapy in patients with HeartWare ventricular assist device thrombosis: a single-institutional experience

**DOI:** 10.1093/icvts/ivac054

**Published:** 2022-03-02

**Authors:** Henrik Fox, Takayuki Gyoten, Sebastian V Rojas, Volker Lauenroth, Sabina Günther, René Schramm, Jan F Gummert, Michiel Morshuis

**Affiliations:** 1 Clinic for Thoracic and Cardiovascular Surgery, Herz- und Diabeteszentrum NRW, Ruhr-Universität Bochum, Bad Oeynhausen, Germany; 2 Heart Failure Department, Herz- und Diabeteszentrum NRW, Ruhr-Universität Bochum, Bad Oeynhausen, Germany

**Keywords:** Left ventricular assist devices, Thrombolysis, Pump thrombosis, Tissue plasminogen activator, Surgical pump exchange, HeartWare

## Abstract

**OBJECTIVES:**

Pump thrombosis remains a major challenge in heart failure patients with left ventricular HeartWare assist device. Current International Society for Heart and Lung Transplantation recommendations favour surgical pump exchange over lysis because safety and efficacy of lysis has been controversially reported. This study summarizes our experience on our HeartWare thrombosis prevention strategy as well as thrombolysis through implementation of our institutional standardized HeartWare assist device protocol.

**METHODS:**

Outcomes of all HeartWare thrombosis patients admitted between 2010 and 2020 were analysed. Thrombolysis therapy using tissue plasminogen activator was used as the first-line therapy in this study and thrombolysis therapy efficacy was defined as freedom from stroke, bleeding, recurrent HeartWare assist device thrombosis or surgical device exchange within 30 days after lysis application.

**RESULTS:**

A total of 507 patients have been included in this study and 66 patients (13%) collectively developed a first HeartWare-thrombosis after a median of 12 months (8–22 months) after HeartWare implantation. Forty patients were treated with unstandardized lysis, of whom 7 patients had thrombolysis associated complications, such as incomplete thrombus resolution requiring surgical pump exchange in 4 patients, but also intracranial haemorrhage occurring in 3 patients. Three patients died in the non-protocol group. Eight device thrombosis patients were treated according to our protocol, showing no lysis-associated complication.

**CONCLUSIONS:**

Despite current recommendations, preferring surgical HeartWare pump exchange in thrombosis, thrombolysis therapy for first HeartWare thrombosis can be safe and effective in a standardized protocol setting, including anticoagulation adjustment and intensified blood pressure control management.

**Figure:**
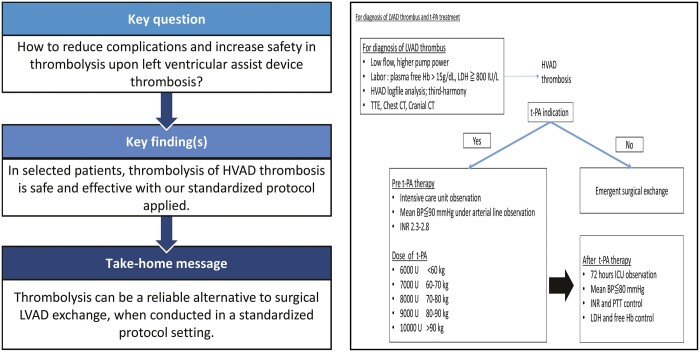

## INTRODUCTION

Long-term mechanical circulatory support using left ventricular assist devices (LVAD) has increasingly become an established treatment option in advanced heart failure (HF) with improved outcomes during the past 2 decades [1, 2]. In clinical routine, indication for LVAD implantation is driven by progressive HF with severely impaired left ventricular function when medical and device-based therapies are exhausted [1, 2]. However, after LVAD implantation, LVAD thrombus formation either in the inflow cannula, in the device body or in the outflow graft is a feared serious complication that can result in life-threatening low-flow conditions or even pump stop, implicating haemodynamic instability, haemolysis, as well as a renal or hepatic failure but also cerebral or peripheral thromboembolism [1–4].

Incidence of LVAD thrombosis in modern continuous-flow LVAD (cf-LVAD) has been reported to emerge in 2–13% of adult LVAD patients [4–6]. However, treatment options for cf-LVAD thrombosis are still under lively discussion and the 2020 International Society for Heart and Lung Transplantation (ISHLT) recommendations on cf-LVAD thrombosis endorse surgical LVAD exchange as the therapy of first choice [7]. While LVAD exchange is associated with invasiveness and a re-do procedure, connoting prolonged recovery periods and the risk of remaining detriments such as infection, organ injury or failure [4, 8], in cases of high perioperative risk or to avoid re-do procedures in patients awaiting heart transplantation, thrombolysis therapy with tissue plasminogen activator (t-PA) may be an alternative [7]. It is well known that t-PA therapy can be associated with bleeding incidence and uncoupling thrombotic events, why t-PA is considered less effective and of high bleeding risk [9]. However, because currently mainly individual case-by-case decisions had led the way, recently published literature proposed improved strategies, not only to ascertain diagnosis but also to employ advanced lysis managements, through the implementation of uniform protocols. In addition to that, although the HeartWare LVAD (HVAD) is not commercially available anymore, numerous patients are implanted with an HVAD and remain in clinical follow-up, why the topic of addressing HVAD pump thrombosis continues to be of clinical relevance. Moreover, the development of HVAD pump thrombosis is associated with worse long-term outcomes, why we both report short- and long-term results [7].

Therefore, with this study, we report our short- and long-term results on using t-PA as the first-line therapy in HVAD thrombosis with implementation of a standardized, institutional protocol [institutional standardized HVAD pump thrombosis lysis protocol (ISHP)].

## METHODS

### Study design

This is a single-center observational cohort study to investigate the safety, efficacy and outcomes of lysis therapy in HVAD pump thrombosis before and after implementation of our ISHP.

### Ethics statement

This single-centre study was approved by the institutional ethics committee of Ruhr-University (2020-619).

### Data collection and follow-up

We included all patients at our institution undergoing HVAD thrombolysis or surgical HVAD exchange between 2010 and 2020. Clinical decisions were made in interdisciplinary heart team conferences, comprised of cardiologists, cardiac surgeons, perfusionists, cardio-anesthesiologists, psychologists and ventricular assist device (VAD) coordinators. Patients receiving biventricular assist devices or patients younger than 18 years of age were excluded from this study. Thrombolysis using t-PA has been the first-choice therapy in this study to treat first HVAD thrombosis, while surgical HVAD exchange was still a possible decision in individual clinical settings. Efficacy of t-PA therapy was defined as no remaining HVAD thrombus proof, normalization of laboratory parameters as well as normalized HVAD log-readout and function. Thrombolysis success was defined as freedom from further thrombolysis events, surgical HVAD exchange and mortality 30 days after t-PA application. Study end-point additionally includes all-cause mortality and the necessity of heart transplantation during follow-up for any HVAD-related complication.

### Diagnosis of HeartWare left ventricular assist device thrombosis and conductance of lysis therapy

**Figure 1:**
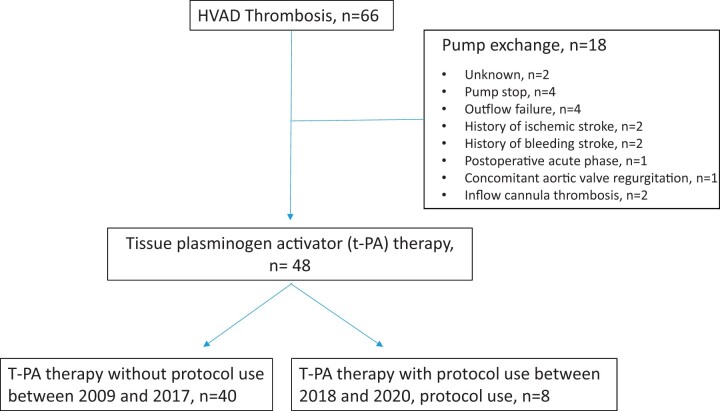
Study flow chart of HeartWare left ventricular assist device thrombosis patients and reasons for surgical pump exchange. Number of patients with tissue plasminogen activator lysis treatment with and without standardized tissue plasminogen activator thrombolysis protocol.

**Figure 2:**
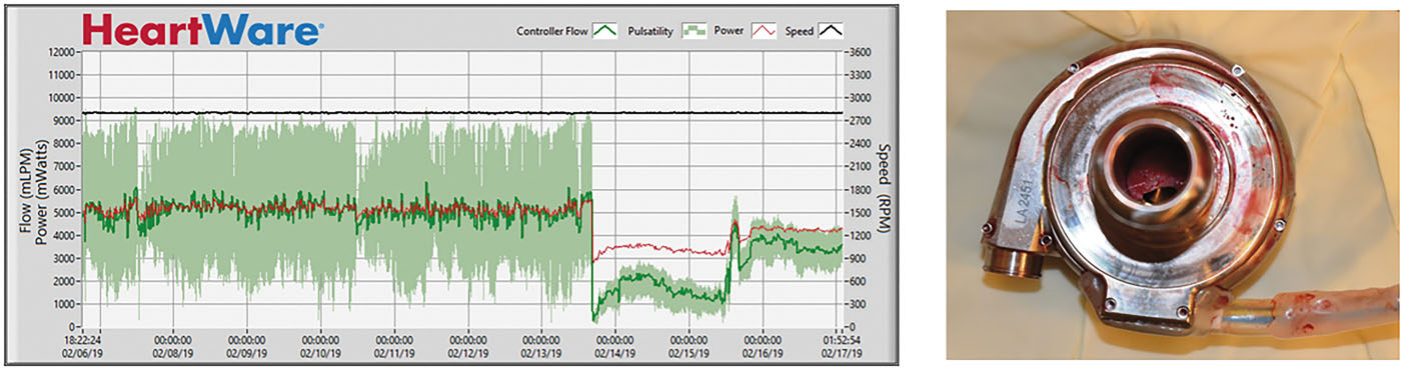
HeartWare left ventricular assist device readout with a typical log file of HeartWare left ventricular assist device thrombosis (left) with flow decrease. On the right-side intraoperative findings of HeartWare left ventricular assist device thrombosis with intra-cannula thrombus localization.

When HVAD thrombosis was suspected in clinical settings for progressive HF symptoms, HVAD low-flow alarm, with no improvement for fluid compensation, but elevated laboratory parameters, including free haemoglobin, lactate dehydrogenase (LDH) and systemic haemoglobin descend, patients qualified for lysis therapy. Moreover, imaging diagnostics were performed, including transthoracic echocardiography and contrast dye computed tomography. Thresholds for HVAD thrombosis diagnosis were defined for LDH above 800 units per litre and a quantitative plasma-free haemoglobin of >15. Suspicious HVAD log file readouts included abnormal characteristics such as ‘third-harmony’, which is an established parameter indicating impeller imbalance, in addition to noteworthy wave patterns in HAVD thrombosis events (Figs 1 and 2).

Although there is evidence that intracardiac lysis therapy may be beneficial in HVAD pump thrombosis, with present data available, only systemic thrombolysis therapy in HVAD pump thrombosis has been investigated in this trial.

### Proposed HeartWare left ventricular assist device thrombosis institutional protocol for standardized conductance of lysis therapy

**Figure 3:**
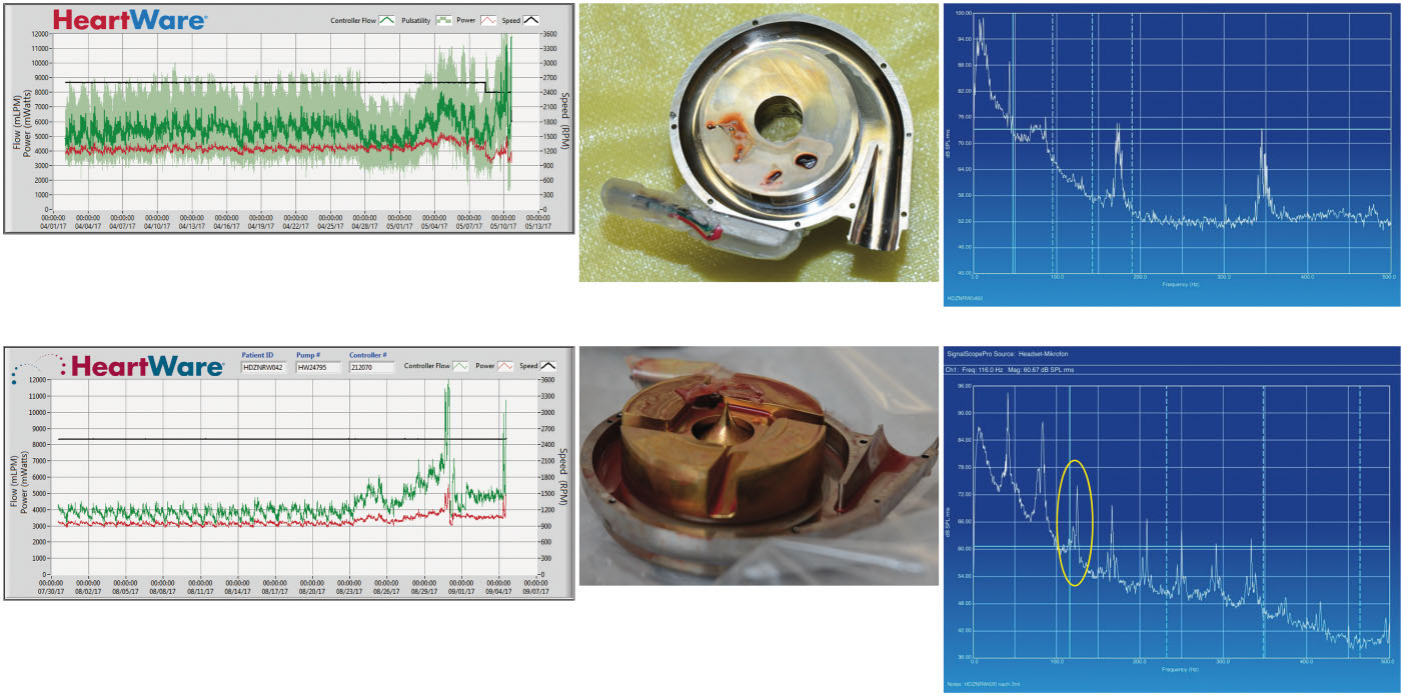
HeartWare left ventricular assist device readout with log file of HeartWare left ventricular assist device thrombosis with increased power consumption and flow changes (left), intraoperative findings of HeartWare left ventricular assist device thrombosis with HeartWare left ventricular assist device intra-corpus thrombus localization (middle), HeartWare left ventricular assist device readout with typical ‘third harmony’.

Our protocol was implemented in 2018 and all consecutive patients have been treated according to this protocol, which implies not only continuous patient vital sign surveillance and continuous monitoring on the intensive care unit but also defined target values for blood pressure and international normalized ratio (INR) adjustments with predefined target values as the goal of therapy. Within our protocol, all patients received continuous intensive care observation and all patients were equipped with invasive haemodynamic monitoring, where mean blood pressure has been appointed to no more than 60 mmHg. INR target range is between 2.3 and 2.8 for the protocol, and INR must meet this range before t-PA application. Heparin was used if the target INR was below pursued therapeutic INR range. Dosages of t-PA used are depicted in Fig. 3 adapted to patient’s weight. Before t-PA application, routinely, cranial computed tomography was performed in all patients to identify contraindications of t-PA application, such as cerebrovascular incidents, intracranial bleeding or recent stroke. Patients stayed in intensive care for 3 days (72 h) when t-PA was applied.

Moreover, all patients receive neurology workup and neurology testing that were annually repeated to identify and follow any curtailments. Patients were discharged from intensive care only after clinical relief from augmented HF symptoms and after normalization of laboratory parameters including haemoglobin, free haemoglobin, LDH as well as normalization of HVAD logfile readouts.

**Figure 4:**
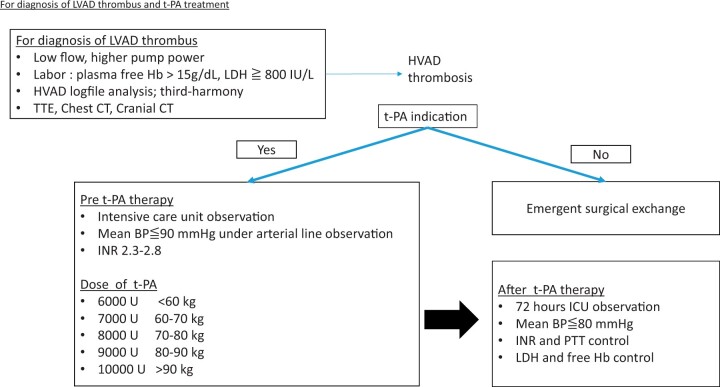
Study protocol and standardized tissue plasminogen activator thrombolysis protocol used in this study since 2018 showing decision tree for treatment applied in this study. BP: blood pressure; CT: computed tomography; Hb: haemoglobin; HVAD: HeartWare left ventricular assist device; ICU: intensive care unit; INR: international normalized ratio; IU: international unit; LDH: lactate dehydrogenase; LVAD: left ventricular assist device; PTT: partial thromboplastin time; t-PA: Tissue plasminogen activator; TTE: transthoracic echocardiogram.

When HVAD thrombosis diagnosis was confirmed, t-PA treatment (intravenous appliance) was administered with vital signs continuously monitored on intensive care unit. Laboratory parameters INR, PTT, LDH and free haemoglobin were regularly rechecked. In patients with haemodynamic instability or insufficient thrombolysis therapy, the heart team was individually consulted to consider surgical HVAD exchange. Although t-PA treatment was the first-line therapy in this study, surgical HVAD exchanges have been performed in haemodynamically unstable patients, at HVAD pump stop and in patients with contraindications for t-PA treatment, such as patients in a perioperative setting of previous surgery, bleeding or stroke. Interdisciplinary heart team was individually consulted to decide on optimal therapy in every case. A graphical description of our ISHP is provided in Fig. 4.

### Statistical analysis

Results are expressed as mean standard deviation in parentheses or as median + 25th–75th percentile interquartile range (IQR) for continuous variables, and frequency and percentage for categorical variables. Kolmogorov–Smirnov test has been used to check for normality. Univariable comparisons were performed with Student’s *t*-test for continuous normally distributed data and group data comparison. The Mann–Whitney *U*-test and Wilcoxon signed rank-sum test were used for comparisons of non-parametric continuous data and Fisher’s exact test for categorical data. Rates of freedom from all-cause death and heart transplantation were generated using the Kaplan–Meier method, and comparisons were made using the stratified log-rank test. A *P*-value of <0.05 was considered statistically significant, and all reported *P*-values are two-sided. All statistical analyses were performed using R (The R Project for Statistical Computing; The R Foundation).

## RESULTS

We included a total of 507 patients in this study, all implanted with cf-LVAD (HeartWare HVAD, Medtronic, USA). This study focused on short-term outcome of HVAD thrombosis therapy regarding bleeding, stroke, death and surgical revision as major short-term outcome parameters. Nonetheless, long-term outcome, including mortality, is additionally included because any HVAD thrombosis event is associated with impaired long-term outcome in the literature.

Baseline characteristics of all HVAD study patients at the time of HVAD implantation are depicted in Table 1. In total, 13% of HVAD patients developed HVAD thrombosis (*n* = 66) after a median of 12 months (IQR; 8–22 months).

**Table 1:** Baseline characteristics of our study patients

	All patients (*n* = 48)	Protocol use (*n* = 8)	Non-protocol use (*n* = 40)	*P*-Value
Age, years, median (IQR)	53 (42–60)	51 (48–59)	54 (38–60)	0.95
Sex, male	46	8	38	>0.999
BSA, median (IQR)	2.03 (1.85–2.15)	2.01 (1.97–2.12)	2.03 (1.84–2.16)	0.96
BMI, median (IQR)	24.9 (22.9–29.3)	24.8 (24.5–25.2)	25.4 (22.8–29.6)	0.78
COPD	5	1	4	>0.999
Diabetes mellitus	11	2	9	>0.999
PAD	4	0	4	>0.999
Pathology				
DCM	11	1	10	0.66
ICM	27	6	21	0.44
BTT	44	8	36	>0.999
History of intracranial Haemorrhage	0	0	0	>0.999
History of ischaemic stroke	11	2	9	>0.999
INTERMACS level, median (IQR)	2 (1.8–3)	2 (1–3)	2 (2–3)	0.60
Laboratory after HVAD implantation, median (IQR)				
LDH	253 (230–313)	243 (231–324)	253 (231–309)	0.80
Free haemoglobin	8 (5–11)	9 (6.5–11.5)	8 (5.0–10.0)	0.48
Thrombocyte	324 (254–395)	308 (224–334)	338 (256–409)	0.13
INR	2.6 (2.3–2.7)	2.7 (2.7–3.1)	2.5 (2.3–2.7)	0.069
Pump information (log file readout), median (IQR)				
Pump power	4.2 (3.7–4.7)	4.2 (3.8–4.7)	4.1 (3.6–4.7)	0.74
Pump speed	2660 (2600–2800)	2700 (2600–2700)	2660 (2600–2800)	0.75

BMI: body mass index; BSA: body surface area; BTT: bridge to transplantation indication; COPD: chronic obstructive pulmonary disease; DCM: dilatative cardiomyopathy; HVAD: HeartWare left ventricular assist device; ICM: ischaemic heart failure; INR: international normalized ratio; INTERMACS: Interagency Registry for Mechanically Assisted Circulatory Support; IQR: interquartile range; LDH: lactate dehydrogenase; PAD: peripheral artery disease.

HVAD readouts and laboratory parameters at HVAD thrombosis events are summarized in Table 2. Baseline characteristics at the time of HVAD implantation of the 48 patients who underwent t-PA treatment in this study are depicted in Table 1. HVAD logfile readouts and laboratory parameters at the time of HVAD thrombosis are summarized in Table 2, including clinical outcomes. The majority of cases had the typical logfile readout with a combination of low flow, increased pump power consumption and pulsatility (Table 2). At all times, blood coagulation prothrombin time at the time of HVAD thrombosis measured through INR measurement was well controlled (IQR; INR 2.2–2.7). Eighteen patients underwent surgical HVAD exchange for t-PA contraindications (Fig. 4).

**Table 2:** Clinical and laboratory characteristics of our HeartWare left ventricular assist device thrombolysis patients at the time of HeartWare left ventricular assist device thrombosis including treatment outcome

Variable at thrombosis	All patients (*n* = 48)	Protocol use	Non-protocol use	*P*-Value
Time between HVAD and 1st event (months), median (IQR)	12 (9-22)	12 (9-21)	18 (11-39)	0.19
Mean blood pressure at event <80 mmHg	14	2	12	>0.999
Laboratory at 1st thrombosis, median (IQR)				
LDH	1480 (1007–2175)	727 (651–1085)	1530 (1160–2340)	0.017
Free haemoglobin	86 (37–188)	50 (31–102)	93 (37–213)	0.18
Thrombocytes	18.1 (13.5–22.4)	18.7 (13.3–22.2)	18.0 (13.9–22.1)	0.80
INR	2.5 (2.2–2.7)	2.6 (2.2–2.8)	2.5 (2.2–2.7)	0.85
Creatinine	1.6 (1.4–2.2)	1.6 (1.4–2.2)	1.5 (0.98–2.0)	0.25
Total bilirubin	0.71 (0.48–1.0)	0.83 (0.54–0.97)	0.68 (0.47–1.0)	0.70
Pump information, median (IQR)				
Pump power	4.8 (4.8–7.0)	5.1 (4.5–5.7)	5.7 (4.9–7.1)	0.22
Pump speed	2700 (2600–2800)	2700 (2700–2750)	2700 (2600–2800)	0.95
Peri-therapeutic outcomes				
Efficacy	41	8	33	0.58
Peri-therapeutic death	3	0	3	
Subsequent pump exchange due to residual/recurrent thrombosis	4	0	4	
ICB	3	0	3	>0.999
Follow-up				
Recurrent pump thrombosis	19	2	17	0.249

HVAD: HeartWare left ventricular assist device; ICB: intracranial bleeding; INR: international normalized ratio; IQR: interquartile range; LDH: Lactate dehydrogenase.

### Clinical short- and long-term outcome results of thrombolysis therapy in the—before protocol implementation—group

Forty patients had unstandardized individual thrombolysis therapy using t-PA at individual physician’s discretion with a t-PA success rate of 83.3%. During follow-up, 22 patients (55%) remained free from further thrombosis events over 48 months of follow-up in this study (IQR; 37–66 months). Recurrent HVAD thrombosis occurred in 16 patients (40%) after a median of 5 months follow-up (IQR; 3–8.8 months). In these 16 patients, heart transplantation, HVAD pump exchange and re-thrombolysis therapy were performed at respective individual decision.

**Figure 5:**
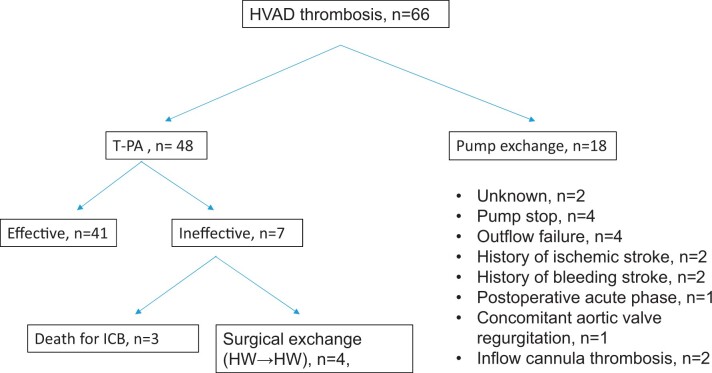
Flow charts of all HeartWare left ventricular assist device thrombosis patients, including outcome of treatment including follow-up. HVAD: HeartWare left ventricular assist device; HW: HeartWare; ICB: intracranial bleeding; t-PA: tissue plasminogen activator.

Seven patients (17.5%) had insufficient thrombolysis results and 3 patients developed severe intracerebral bleeding, concluding in death (Table 2). The other 4 ineffective t-PA patients underwent subsequent surgical HVAD exchange. None of the surgical HVAD exchange patient developed recurrent HVAD thrombosis during the follow-up period of this study (IQR; 10–19 months). According to Kaplan–Meier analysis for all-cause death, the overall survival at 12 months after effective t-PA thrombolysis was 87.5% (95% CI; 74.3–94.2%). Fig. 5 summarizes outcomes.

### Clinical short- and long-term outcome results of thrombolysis therapy in the—after protocol implementation (institutional standardized HeartWare left ventricular assist device pump thrombosis lysis protocol)—group

Because of the high complication rates, we developed our ISHP to consistently manage HVAD t-PA therapy, aiming to reduce complication rates and adverse events. Since 2018, all HVAD thrombolysis treatments were conducted in accordance with our ISHP only. Therefore, 8 patients received t-PA therapy for HVAD thrombosis after implementation of our ISHP in this study. Patients treated with ISHP had statistically significant higher LDH values than patients treated with unstandardized, individual t-PA therapy (*P* = 0.024). Importantly, all 8 patients had effective HVAD thrombolysis (100%) and all patients have been discharged without further complications. Two patients experienced recurrent HVAD thrombosis at 2 and 3 months after ISHP therapy during study follow-up, and 1 patient effectively had re-t-PA treatment without any further complication and without further HVAD thrombosis throughout study follow-up. The second patient also had re-t-PA therapy, which was ineffective, and this patient received heart transplantation.

### Clinical results in leftover patients of surgical HeartWare left ventricular assist device exchange because of contraindications for tissue plasminogen activator application

Eighteen patients (27%) who were ineligible for ISHP practice, mainly for unstable haemodynamics and contraindications for t-PA therapy, were treated with surgical HVAD exchange subsequently. Moreover, thrombus localization in these cases mainly was inside the outflow graft (*n* = 4). Additional reasons for surgical HVAD exchange were HVAD pump stop and cardiac resuscitation (*n* = 4). Surgical HVAD exchange was performed in 15 of the 18 patients, but 3 patients received a device exchange into HeartMate3 (Abbott, IL, USA). According to all-cause death Kaplan–Meier analysis, overall survival at 3 and 12 months after surgical HVAD exchange was 72.2% (95% CI; 45.6–87.4%) and 60.2% (95% CI; 34.0–78.7%), respectively.

### Survival analysis

Survival after t-PA thrombolysis at 3 months was 83.3% (95% CI; 69.4–91.3%) over 72.2% (95% CI; 45.6–87.4%) for surgical HVAD exchange. Twelve-month survival was 70.3% (95% CI; 55.0–81.2%) in t-PA over 60.2% (95% CI; 34.0–78.7%) after surgical HVAD exchange, while the decision for each therapeutic approach had been made individually.

## DISCUSSION

We report the largest available single-centre trial to investigate safety and efficacy and also the outcome of thrombolysis treatment in patients with HVAD pump thrombosis so far. Through the implementation of our ISHP, feared major lysis complications have been significantly reduced. With this approach, HVAD thrombolysis appears to be a safe and effective alternative over surgical HVAD exchange for the treatment of first HVAD pump thrombosis, when applied in a standardized setting.

HVAD pump thrombosis remains a feared and clinically challenging complication, even in patients with well controlled INR, as described before [4]. Current scientific discussion about the optimal therapeutic approach to handle HVAD pump thrombosis is vitally ongoing and therapy decision either for surgical pump exchange or thrombolysis therapy is often made on an individual preferences [1]. Many physicians refrain from thrombolysis application because they fear bleeding complications that can become uncontrollable [7, 10]. Therapy standards to enhance the safety and efficacy of thrombolysis in HVAD patients are scarcely investigated so far, why it is interesting to study the potential, facilities, efficacy and safety of thrombolysis therapy in more detail, to improve outcome HVAD thrombosis patients.

In our trial, HVAD thromboses mainly occurred within the first 12 months after HVAD implantation. Three patients died for severe intracerebral haemorrhage when treated with unstandardized t-PA thrombolysis before we had implemented our ISHP, and 2 patients developed recurrent HVAD thrombosis after thrombolysis during study follow-up. One patient required heart transplantation for a recurrent HVAD thrombosis with ineffective thrombolysis.

In our study, effectiveness and complications of thrombolysis therapy have been analysed in HVAD patients with first thrombosis using systemic t-PA to test efficacy and safety through illustration of feared prompt complications. Surprisingly and in contrast to current ISHLT recommendations, our results encourage thrombolysis use, because we can demonstrate less complications, more effective but also safe thrombolysis management through implementation of our ISHP. Moreover, our small study appears to show hints of improved survival in HVAD thrombosis patients treated with t-PA thrombolysis over surgical HVAD exchange in mortality analysis. Yet, for lack of statistical power, this study cannot substantiate the superiority of our ISHP through statistical analyses for all important issues in the field, but our findings encourage implementation of a standardized operating procedural protocol to manage HVAD thrombosis, as an alternative to ISHLT recommended surgical HVAD exchange.

In the current literature, it is believed that surgical HVAD exchange is the treatment of choice and that surgical HVAD exchange would be safer and more effective over any thrombolysis treatment [1, 2, 7]. However, perioperative mortality and morbidity appears high after surgical HVAD exchange, and long-term follow-up outcomes after surgical exchange have not been sufficiently studied yet [7]. Surgical pump exchange is associated with additional potential complications such as postoperative ventricular tachycardia, right HF and HVAD infection, as well as impaired quality of life for wound infection and immobility [7]. Moreover, in a bridge-to-transplantation indication, resternotomy may make subsequent heart transplantation more difficult due to augmented adhesions through an additional surgical procedure [6, 11]. In haemodynamically unstable patients, surgical HVAD exchange is considered the preferred method to overrule hypoperfusion and to prevent life-threatening HVAD pump stop, but no trial has shown the superiority of any approach in such patients so far.

In a multicentre analysis of 21 HVAD thrombosis patients, t-PA therapy was associated with an effectiveness of only 48% and a high rate of complications such as haemorrhagic stroke (21%) and death (10%) [9]. Therefore, this trial favoured surgical HVAD exchange as the treatment of choice, even in patients with stable haemodynamics [9]. Contrary to this, the Interagency Registry for Mechanical Assisted Circulatory Support report states surgical HVAD exchange to be associated with increased mortality and impaired prognosis, with a 1-year survival of 65% [12]. Furthermore, 1-year survival is even worse in patients with a second HVAD exchange procedure for recurrent HVAD thrombosis [12].

While in this context improved management is warranted, thrombolysis therapy may represent a safe and effective alternative, and associated complications may be reduced through implementation of specific protocols as suggested before [13]. To this effect, some authors report HVAD thrombolysis to then be superior over surgery if applied in a standardized setting in selected patients [14]. This is in line with a 2016 study of 50 patients of Oezpeker *et al.* [15], favouring systemic thrombolysis therapy over surgical HVAD exchange. In our study, thrombolysis efficacy was 85%, which is a higher efficacy rate than what is published in available reports to date [13]. Before ISHP implementation, we identified 7 patients at our institution with ineffective t-PA and 3 patients died of intracerebral haemorrhage after thrombolysis, which is a particularly feared t-PA complication [10]. This points out the importance to optimize treatment and management through ISHP exercise including tangible diagnosis and improved surveillance to prevent critical thrombolysis incidences.

Our protocol particularly addresses 2 aspects: first is exact thrombus localization, which is of essential value to achieve effective t-PA therapy, because inflow cannula thrombosis more often results in haemodynamic instability (Fig. 5). On the other hand, outflow cannula thrombus localization is often associated with additional pathologies, such as outflow graft kinking and increased mechanical pressure from the driveline, and all such aspects cannot be addressed through t-PA, why these settings are prone to develop recurrent HVAD thrombosis afterwards.

Our 48 HVAD thrombosis patients mainly had thrombus localization inside the device body and identified through HVAD log-file readouts. The effectiveness of thrombolysis therapy was 85%, resulting in log-file normalizations.

Second, our protocol strictly appoints target values to control blood pressure and vital sign parameters based on invasively measured haemodynamics that were continuously monitored on intensive care unit. Interestingly, 7 patients with ineffective t-PA therapy all had their event before our ISHP implementation in 2018 while since ISHP exercise no complications in the context of t-PA therapy were observed. Although statistical power is limited for the small number of patients available for this trial, our findings suggest not only increased t-PA lysis efficacy but also safety.

Herein, in particular, increased blood pressure is an accepted risk factor for intracerebral haemorrhage [10] and since ISHP implementation at our institution, no intracranial complication occurred even during follow-up.

In addition to that, increased blood pressure is not only associated with periprocedural t-PA complications, but it is also an established high-risk factor for HVAD thrombosis development *per se*. Therefore, our study patients had strict blood pressure targets during t-PA therapy application within the protocol, resulting in less complications during thrombolysis. Moreover, these strict defined blood pressure targets in the subsequent outpatient setting may have contributed to the observed decreased risk of HVAD thrombosis development during follow-up additionally.

Beyond all, there are numerous other factors that are associated with HVAD thrombosis development that our study has not been able to address, why larger studies in a randomized, controlled, clinical setting are needed to confirm our encouraging findings but also to investigate additional influencing topics for this particular patient population.

### Limitations

Although our study is the largest single-centre study to report safety and efficacy through implementation of a standardized institutional HVAD t-PA thrombolysis therapy protocol so far, there are several limitations to our study. This is a single-centre study with a limited number of patients and the study may have missed additional seminal factors contributing to HVAD pump thrombosis. The study has not a randomized, controlled design, why additional confounding factors or influencing aspects may have been missed. Therefore, our study findings do not allow for generalized statements, but still we are able to report encouraging outcomes in this rarely investigated cohort. This study is also meant to inspire future studies to investigate our hypotheses in more detail.

## CONCLUSION

Despite current ISHLT recommendations, preferring surgical HVAD pump exchange in HVAD thrombosis, t-PA thrombolysis therapy for first HVAD thrombosis can be safe and effective in a standardized protocol setting, including anticoagulation adjustment and intensified blood pressure control management.

**Conflict of interest:** none declared.

## Data Availability Statement

All relevant data are within the manuscript and its supporting information files.

## Author contributions

**Henrik Fox:** Conceptualization; Investigation; Methodology; Project administration; Validation; Writing—original draft; Writing—review & editing. **Takayuki Gyoten:** Data curation; Formal analysis; Investigation; Methodology; Visualization. **Sebastian V. Rojas:** Project administration; Resources; Supervision; Validation; Writing—review & editing. **Volker Lauenroth:** Data curation; Investigation. **Sabina Günther:** Formal analysis; Funding acquisition; Supervision; Writing—review & editing. **René Schramm:** Validation; Visualization; Writing—original draft; Writing—review & editing. **Jan F. Gummert:** Project administration; Resources; Supervision. **Michiel Morshuis:** Data curation; Funding acquisition; Investigation; Methodology; Supervision; Writing—review & editing.

## Reviewer information

Interactive CardioVascular and Thoracic Surgery thanks Carlos A. Mestres, Roberto Lorusso and the other, anonymous reviewer(s) for their contribution to the peer review process of this article.

## ABBREVIATIONS

cf-LVAD: Continuous-flow LVAD
HF: Heart failure
HVAD: HeartWare LVAD
ISHP: Institutional standardized HVAD pump thrombosis lysis protocol
INR: International normalized ratio
ISHLT: International Society for Heart and Lung Transplantation
IQR: Interquartile range
LDH: Lactate dehydrogenase
LVAD: Left ventricular assist devices
t-PA: Tissue plasminogen activator
